# Exploring the Potential of *Corynebacterium glutamicum* to Produce the Compatible Solute Mannosylglycerate

**DOI:** 10.3389/fbioe.2021.748155

**Published:** 2021-09-21

**Authors:** Andreas Schwentner, Heiko Neugebauer, Serin Weinmann, Helena Santos, Bernhard J. Eikmanns

**Affiliations:** ^1^ Institute of Microbiology and Biotechnology, Ulm University, Ulm, Germany; ^2^ Instituto de Tecnologia Química e Biológica António Xavier, Universidade Nova de Lisboa, Oeiras, Portugal

**Keywords:** mannosylglycerate, compatible solute, metabolic engineering, Corynebacterium glutamicum, bacterial milking, mannose metabolization, bifunctional mannosylglycerate synthase

## Abstract

The compatible solute mannosylglycerate (MG) has exceptional properties in terms of protein stabilization and protection under salt, heat, and freeze-drying stresses as well as against protein aggregation. Due to these characteristics, MG possesses large potential for clinical and biotechnological applications. To achieve efficient MG production, *Corynebacterium glutamicum* was equipped with a bifunctional MG synthase (encoded by *mgsD* and catalyzing the condensation of 3-phosphoglycerate and GDP-mannose to MG) from *Dehalococcoides mccartyi*. The resulting strain *C. glutamicum* (pEKEx3 *mgsD*) intracellularly accumulated about 111 mM MG (60 ± 9 mg g_CDW_^−1^) with 2% glucose as a carbon source. To enable efficient mannose metabolization, the native *manA* gene, encoding mannose 6-phosphate isomerase, was overexpressed. Combined overexpression of *manA* and *mgsD* from two plasmids in *C. glutamicum* resulted in intracellular MG accumulation of up to ca. 329 mM [corresponding to 177 mg g _cell dry weight (CDW)_^−1^] with glucose, 314 mM (168 mg g_CDW_^−1^) with glucose plus mannose, and 328 mM (176 mg g_CDW_^−1^) with mannose as carbon source(s), respectively. The product was successfully extracted from cells by using a cold water shock, resulting in up to 5.5 mM MG (1.48 g L^−1^) in supernatants. The two-plasmid system was improved by integrating the *mgsD* gene into the *manA*-bearing plasmid and the resulting strain showed comparable production but faster growth. Repeated cycles of growth/production and extraction of MG in a bacterial milking-like experiment showed that cells could be recycled, which led to a cumulative MG production of 19.9 mM (5.34 g L^−1^). The results show that the newly constructed *C. glutamicum* strain produces MG from glucose and mannose and that a cold water shock enables extraction of MG from the cytosol into the medium.

## Introduction

Compatible solutes are compounds of low molecular weight that are readily accumulated in the cytoplasm of microorganisms up to molar concentrations as a reaction to increasing extracellular osmolality (Santos and da Costa, 2001; Santos et al., 2006). Particularly, microorganisms adapted to extreme environments, such as hyperthermophiles and halophiles, accumulate compatible solutes not only as a response to salt stress but also in response to supraoptimal growth temperatures (Silva et al., 1999; Esteves et al., 2014). In contrast to compatible solutes from mesophilic organisms, which commonly accumulate neutral and nitrogen-containing compounds, such as L-proline, betaine, or ectoine, compatible solutes from (hyper)thermophilic organisms commonly are negatively charged and carbohydrate-based (Borges et al., 2014). One of the most widespread compatible solutes in (hyper)thermophilic microorganisms is α-D-mannopyranosyl-(1--˃2)-D-glycerate, also known as mannosylglycerate (MG), digeneaside, or firoin (Empadinhas and da Costa, 2008; Borges et al., 2014).

Most known native producers synthesize MG *via* a two-step pathway in which the enzymes mannosyl 3-phosphoglycerate synthase (MPGS) and mannosyl 3-phosphoglycerate phosphatase (MPGP) subsequently convert guanosine diphosphate mannose (GDP-Man) and 3-phosphoglycerate (3-PG) to MG (Borges et al., 2004; Borges et al., 2014). However, there are also organisms, e.g., the mesophilic bacterium *Dehalococcoides mccartyi (*formerly *D. ethenogenes),* which uses a single-step pathway *via* a bifunctional synthase/phosphatase called mannosylglycerate synthase (MgsD), encoded by *mgsD* and converting 3-PG and GDP-Man to MG (Empadinhas et al., 2004). Heterologous expression of the *mgsD* gene resulted in intracellular accumulation of MG in *Saccharomyces cerevisiae*, confirming the functionality of MgsD of *D. mccartyi* as bifunctional MG synthase/phosphatase (Empadinhas et al., 2004).

MG is a molecule with outstanding characteristics in protein stabilization *in vitro* and *in vivo*, which opens up a vast field of applications (Lentzen and Schwarz, 2006; Jorge et al., 2016). MG was shown to protect model proteins (e.g., lactate dehydrogenase and glucose oxidase; Borges et al., 2002), against heat inactivation and freeze-drying and prevented heat-induced aggregation; it led to enhanced stability of cutinase (Melo et al., 2001), recombinant nuclease (Faria et al., 2004), and retroviral vaccines (Cruz et al., 2006). Strikingly, MG was also effective in suppressing aggregation of soluble β-amyloid peptides into fibrils, one of the key pathological features of Alzheimer’s disease (Ryu et al., 2008). In a yeast model of Parkinson’s disease, MG prevented aggregation of α-synuclein, the major component of the intraneuronal inclusions in the brain of patients suffering from this disease (Faria et al., 2013). These characteristics render MG an attractive target for microbial production.

*Corynebacterium glutamicum* is a Gram-positive, facultatively anaerobic bacterium that natively grows on a variety of sugars, alcohols, organic acids, and other substrates (Becker and Wittmann, 2015). Furthermore, the organism has been engineered to metabolize nonnative carbon sources such as xylose (Kawaguchi et al., 2006; Meiswinkel et al., 2013; Lange et al., 2017), N-acetylmuramic acid (Sgobba et al., 2018), mannitol (Laslo et al., 2012), starch (Seibold et al., 2006), or cellobiose (Adachi et al., 2013). Generally, *C. glutamicum* is well known for the large-scale industrial production of amino acids, mainly L-glutamate and L-lysine (Eggeling and Bott, 2015; Wendisch et al., 2016). During the past decade, however, the range of products has been expanded drastically and comprises nowadays not only other proteinogenic amino acids, such as L-valine (Blombach et al., 2007a; Schwentner et al., 2018), L-arginine (Park et al., 2014), L-tryptophan (Zhang et al., 2015), or L-histidine (Schwentner et al., 2019), but also organic acids (Wieschalka et al., 2013), alcohols (Blombach et al., 2011), carotenoids (Henke and Wendisch, 2019), and proteins (Bakkes et al., 2020; Hemmerich et al., 2020). Moreover, *C. glutamicum* has been engineered to overproduce native compatible solutes such as trehalose (Carpinelli et al., 2006) and L-proline (Jensen and Wendisch, 2013), as well as the nonnative α-D-glucosylglycerol (Roenneke et al., 2018), L-pipecolic acid (Pérez-García et al., 2019), and ectoine/hydroxyectoine (Gießelmann et al., 2019), principally demonstrating the capability of *C. glutamicum* to serve as a host for production of compatible solutes. Very recently, *C. glutamicum* has been also engineered to produce MG from glucose plus glycerate and from mannose plus glycerate in an artificially designed starch-mannose-fermentation biotransformation process, respectively (Tian et al., 2020).

Mannose is a C-2 epimer of glucose, which is widespread in lignocellulosic biomass and makes up 20% of the sugars from biomass hydrolysates of wood (Sasaki et al., 2011). Sasaki and coworkers showed that uptake of mannose in *C. glutamicum* strain R is mainly mediated *via* the glucose-specific phosphoenolpyruvate- (PEP-) dependent phosphotransferase system PtsG, which can be substituted for by the fructose-dependent PtsF in absence of PtsG. The WT strain as well as a *manA* (encoding MPI) overexpressing strain consumed glucose preferentially over mannose when both substrates were present; however, mannose consumption was drastically increased when *manA* was overexpressed. *C. glutamicum* only consumed glucose and mannose simultaneously when *ptsF* was overexpressed. Furthermore, MPI was shown to be essential not only for mannose catabolism but also for the synthesis of GDP-mannose from fructose 6-phosphate (Sasaki et al., 2011).

**FIGURE 1:**
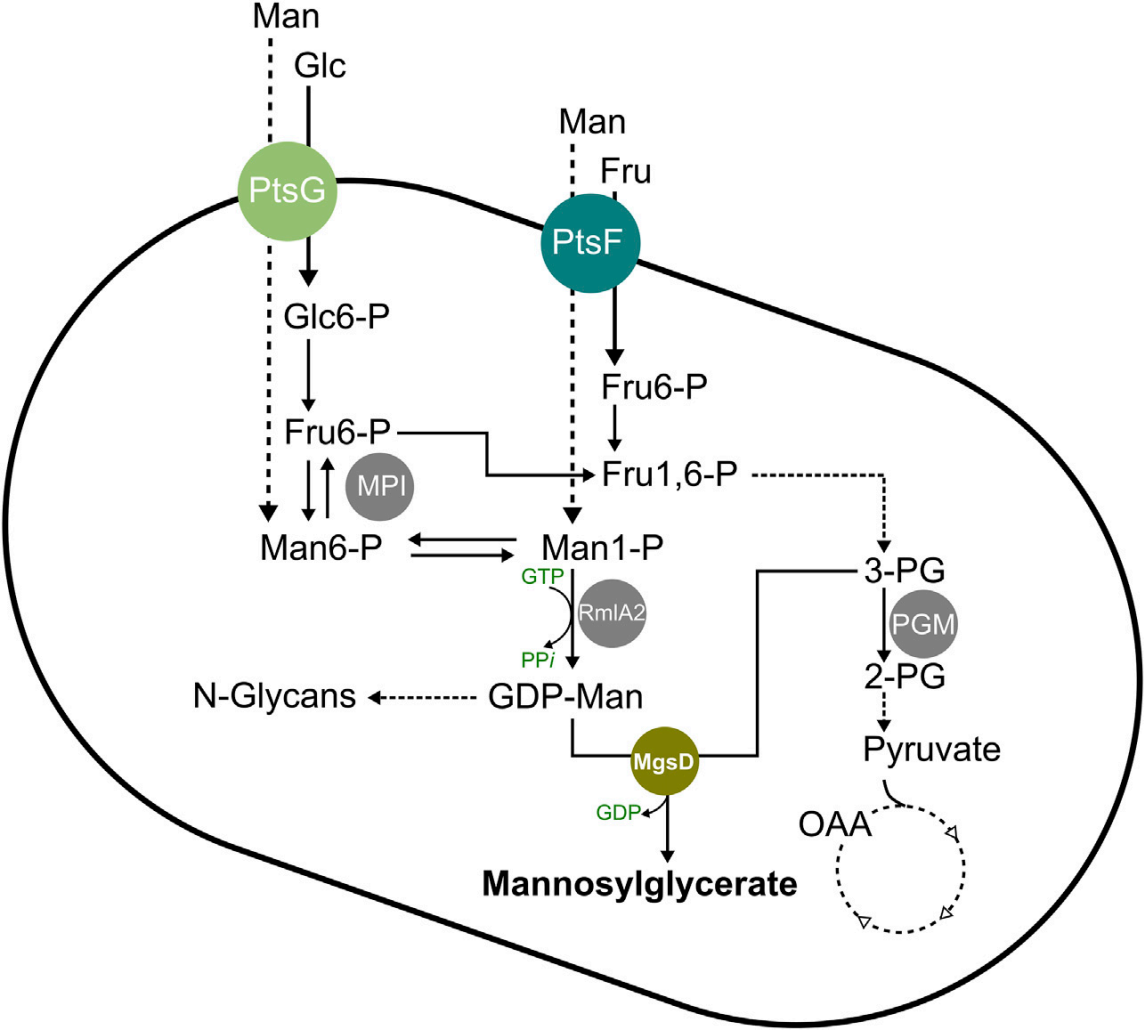
Schematic overview of the mannosylglycerate synthesis pathway in *C. glutamicum*. Abbreviations: 2-PG, 2-phosphoglycerate; 3-PG, 3-phosphoglycerate; Fru, fructose; Fru1,6-P, fructose-1,6-bisphosphate; Fru6-P, fructose 6-phosphate; Glc, glucose; Glc6-P, glucose 6-phosphate; GDP, guanosine diphosphate; GTP, guanosine triphosphate; Man, mannose; Man1-P, mannose 1-phosphate; Man6-P, mannose 6-phosphate; MgsD, mannosylglycerate synthase (encoded by *mgsD* from *Dehalococcoides mccartyi*); MPI, mannose 6-phosphate isomerase (encoded by *manA*); OAA, oxaloacetate; PGM, phosphoglycerate mutase (encoded by *pgm*); PP_*i*_, inorganic pyrophosphate; PtsF, fructose-specific phosphoenolpyruvate-dependent phosphotransferase system (encoded by *ptsF*); PtsG, glucose-specific phosphoenolpyruvate-dependent phosphotransferase system (encoded by *ptsG*); RmlA2, GDP-mannose pyrophosphorylase (encoded by *rmlA*2).

In this study, we aimed to produce the compatible solute MG from glucose with the heterologous host organism *C. glutamicum,* which natively possesses the pathways for the synthesis of the MG precursors GDP-mannose and 3-phosphoglycerate (see Figure 1) by implementing an MG synthase from the native MG producer *D. mccartyi*. The MG-producing *C. glutamicum* was engineered to grow with mannose as a carbon source by overexpressing the native *manA* gene and eventually we produced MG from glucose and/or mannose.

## Materials and Methods

### Bacterial Strains and Plasmids

All bacterial strains, plasmids, and oligonucleotides used in this work and their corresponding characteristics, sequences, and sources are given in Table 1.

**TABLE 1:** Strains, plasmids, and oligonucleotides used in this study.

Strain, plasmid, or oligonucleotide	Relevant characteristic(s) or sequence	Source, reference, or purpose
Strains		
*E. coli* DH5α	F-ϕ80*lacZ*ΔM15 Δ(*lacZYA*-*argF*)U169 *endA1 recA1 hsdR17* (r_K_^−^ m_K_^+^) *supE44 thi-1 gyrA96 relA1 phoA*	Hanahan (1983)
*C. glutamicum* WT	Wild-type (WT) strain ATCC 13032	American Type Culture Collection
*C. glutamicum* (pEKEx3 *mgsD*)	*C. glutamicum* WT carrying the heterologous *mgsD* gene from *Dehalococcoides mccartyi* in plasmid pEKEx3	This work
*C. glutamicum* (pVWEx1 *manA*)	*C. glutamicum* WT carrying the native gene *manA* in plasmid pVWEx1	This work
*C. glutamicum* (*pEKEx3 mgsD*) (*pVWEx1 manA*)	*C. glutamicum* WT carrying the heterologous *mgsD* gene and the native *manA* gene in plasmids pEKEx3 and pVWEx1, respectively	This work
*C. glutamicum* (*pVWEx1 manA mgsD*)	*C. glutamicum* WT carrying *manA* and *mgsD* genes in plasmid pVWEx1	This work
Plasmids		
pEX-K4 *mgsD*	pEX-K4 containing a codon-optimized *mgsD* gene from *D. mccartyi*	Eurofins Genomics GmbH, Ebersberg, Germany
pEKEx2	*E. coli*-*C. glutamicum* shuttle vector, P_*tac*_, *la*I^q^, Km^R^, and pBL1 ori	Eikmanns et al. (1994)
pEKEx3	*E. coli*-*C. glutamicum* shuttle vector, P_*tac*_,*lac*I^q^, Spec^R^, and pBL1 ori	Stansen et al. (2005)
pVWEx1	*E. coli*-*C. glutamicum* shuttle vector, P_*tac*_, *lac*I^q^, Km^R^, and pCG1 ori	Peters-Wendisch et al. (2001)
pEKEx2 *mgsD*	pEKEx2 containing a codon-optimized *mgsD* gene from *D. mccartyi* under control of P_*tac*_	This work
pEKEx3 *mgsD*	pEKEx3 containing a codon-optimized *mgsD* gene from *D. mccartyi* under control of P_*tac*_	This work
pVWEx1 *manA*	pVWEx1 containing the *manA* gene from *C. glutamicum* under control of P_*tac*_	This work
pVWEx1 *manA mgsD*	pVWEx1 *manA* containing a codon-optimized *mgsD* gene from *D. mccartyi* under control of P_*tac*_	This work
Oligonucleotides		
mgsD-2.1	5′-CCT​CCC​GGA​GCG​AAT​CAG​TC-3′	Sequencing of *mgsD* insert in pEKEx2 *mgsD*
mgsD-2.2	5′-CGG​TGG​AGT​AGG​AGT​ATG​TC-3′	Sequencing of *mgsD* insert in pEKEx2 *mgsD*
mgsD-2.3	5′-CAG​ACC​GCT​TCT​GCG​TTC​TG-3′	Sequencing of *mgsD* insert in pEKEx2 *mgsD*
mgsD1	5′- CAC​TCC​CGT​TCT​GGA​TAA​TG-3′	Sequencing of *mgsD* insert in pEKEx3 *mgsD*
mgsD2	5′-ATA​CTC​CAT​GGT​CCG​TGT​TC-3′	Sequencing of *mgsD* insert in pEKEx3 *mgsD*
mgsD3	5′-ATG​GAC​CTC​GCG​AAG​TTC​TC-3′	Sequencing of *mgsD* insert in pEKEx3 *mgsD*
mgsD4	5′- GCT​ACG​GCG​TTT​CAC​TTC​TG-3′	Sequencing of *mgsD* insert in pEKEx3 *mgsD*
manA_fwd	5′-GAC​GGTCT​AGATAA​AGG​AGT​TTT​ATG​GAG​CTA​TTG​GAA​GG-3′	Amplification of *manA* (*Xba*I site underlined)
manA_rev	5′-CAG​CTGGT​ACCCTA​AAC​CCT​AGC​GAG​GAA​TA-3′	Amplification of *manA* (*Kpn*I site underlined)
mgsD-for	5′-TAGGGT​ACCAGG​AGG​ACA​TAC​AAT​GCG​CAT​CGA​GAG​CCT​GCG-3′	Amplification of *mgsD* (*Kpn*I site underlined)
mgsD-rev	5′-GCTGGT​ACCCTT​ACT​CCA​TAG​GCA​GGA​TAA​TG-3′	Amplification of *mgsD* (*Kpn*I site underlined)
mgsD5	5′-GAC​AAC​TAC​ATC​CCT​GG-3′	Sequencing of *mgsD* insert in pVWEx1 *manA mgsD*
mgsD6	5′-CTG​CCG​TGA​TTG​AGA​AG-3′	Sequencing of *mgsD* insert in pVWEx1 *manA mgsD*
mgsD7	5′-CGC​TCC​ACT​AAT​ATC​GTG-3′	Sequencing of *mgsD* insert in pVWEx1 *manA mgsD*

### Media and Cultivation Conditions

*E. coli* DH5α cells were cultivated aerobically in 5 ml 2xYT complex medium (Green and Sambrook, 2012) at 37 ºC in glass test tubes on a rotary shaker at 130 rpm. Cultivation of *C. glutamicum* cells was performed in glass test tubes or baffled shaking flasks at 30°C on a rotary shaker at 130 rpm. Precultivations of *C. glutamicum* cells were done by streaking cell material from a glycerol stock (30% glycerol [v v^−1^]) on 2xYT agar medium, which then was incubated for 2–3 days at 30°C. Inoculation of the first liquid culture was done by transferring a single colony in 5 ml 2xYT medium in 20 ml glass test tubes, which was incubated for about 7 h. Then, the culture was transferred completely into 50 ml 2xYT medium in a 500 ml baffled shaking flask and incubated overnight (about 14 h). The overnight culture was harvested by centrifugation (4.500 × g, 10 min, 4°C) and the resulting cell pellet was washed in 0.9% (w v^−1^) sodium chloride solution, centrifuged again, resuspended in sodium chloride solution, and then used to inoculate 50 ml modified CGXII minimal medium (Eikmanns et al., 1991) in 500 ml baffled shaking flasks to a starting optical density at 600 nm (OD_600_) of 1. The modified CGXII minimal medium contained 5 g (NH_4_)_2_SO_4_ L^−1^, 5 g urea L^−1^, 21 g 3-morpholinopropanesulfonic acid (MOPS) L^−1^, 1 g K_2_HPO_4_ L^−1^, 1 g KH_2_PO_4_ L^−1^, 0.25 g MgSO_4_ L^−1^, and 0.01 g CaCl_2_ L^−1^. Before the medium was autoclaved, the pH was set to 7.4 by adding 5 M KOH and after autoclaving, 16.4 mg FeSO_4_ × 7 H_2_O L^−1^, 10 mg MnSO_4_ × H_2_O L^−1^, 0.2 mg CuSO_4_ L^−1^, 1 mg ZnSO_4_ × 7 H_2_O L^−1^, 0.02 mg NiCl_2_ × 6 H_2_O L^−1^, 0.2 mg biotin L^−1^, and either 10 or 20 g glucose L^−1^ and/or 10 or 20 g mannose L^−1^ (as indicated in the Results section) were added under sterile conditions. The expression of genes under control of the P_*tac*_ promoter was induced by adding 0.5 mM isopropyl β-d-1-thiogalactopyranoside (IPTG) directly before inoculation into the main culture. For strains carrying plasmids, 50 µg kanamycin mL^−1^ or 100 µg spectinomycin mL^−1^ was added to the media where required. For strains carrying two plasmids, the antibiotic concentrations were reduced to 25 µg kanamycin ml^−1^ and 50 µg spectinomycin mL^−1^. Formation of biomass was followed by measuring OD_600_ with a photometer (Ultrospec® 3000 pro, Amersham Pharmacia Biotech Europe GmbH, Freiburg, Germany). Cell dry weight (in g_CDW_ L^−1^) of the biomass was calculated by applying the correlation CDW = OD_600_ × 0.30 g L^−1^ (Blombach et al., 2007b).

Growth rates µ [h^−1^] were calculated by applying linear regressions of ln(OD_600_) plotted over time in h during the exponential growth phase (between 4 and 10 h of incubation after inoculation).

### Recombinant DNA Work

Standardized methods of molecular cloning such as PCR, DNA restriction, and ligation were carried out according to Green and Sambrook (2012). Plasmids were isolated and PCR fragments purified with the E.Z.N.A.® Plasmid Mini Kit I (Omega Bio-Tek, Inc., Norcross, United States) and the NucleoSpin® Gel and PCR Clean-Up Kit (Macherey Nagel GmbH & Co. KG, Düren, Germany), respectively, according to the manufacturer’s instructions. Genomic DNA from *C. glutamicum* was isolated and purified with NucleoSpin Microbial DNA Mini kit (Macherey Nagel GmbH & Co. KG, Düren, Germany) according to the manufacturer’s instructions.

Electrocompetent cells of *E. coli* DH5α and of *C. glutamicum* were prepared as has been described before (Dower et al., 1988; Tauch et al., 2002). Transformation of *E. coli* and *C. glutamicum* with plasmids was performed as described before (Dower et al., 1988; van der Rest et al., 1999). Transformation of *C. glutamicum* included a heat shock for 6 min at 46°C. *E. coli* DH5α and all *C. glutamicum* strains were electroporated with a MicroPulser Electroporator (Bio-Rad Laboratories GmbH, München, Germany) at 2.5 kV with 600 Ω resistance. Enzymes for recombinant DNA work were obtained from Thermo Fisher Scientific Inc. (Darmstadt, Germany) or New England Biolabs GmbH (Frankfurt am Main, Germany). Oligonucleotides used in this work were ordered from biomers.net GmbH (Ulm, Germany) and are listed in Table 1.

A codon-optimized variant of the *mgsD* gene, encoding mannosylglycerate synthase (MgsD) from *D. mccartyi* strain ATCC BAA-2266/KCTC 15142/195 (formerly *D. ethenogenes* strain 195), was deduced from UniProt protein Q3Z6S5 and synthesized commercially and equipped with restriction sites for *Sal*I and *Kpn*I and ligated into pEX-K4 (Eurofins Genomics GmbH, Ebersberg, Germany). *E. coli* DH5α was transformed with the resulting vector pEX-K4 *mgsD* and the plasmid from transformants was isolated and restricted with *Sal*I and *Kpn*I. The *mgsD* fragment was purified and ligated into pEKEx2 as an intermediate step. The sequence identity of the resulting plasmid pEKEx2 *mgsD* was checked by PCR using primers mgsD-2.1, mgsD-2.2, and mgsD-2.3 (Eurofins Genomics GmbH, Ebersberg, Germany). Plasmid pEKEx2 *mgsD* was then restricted with *Sal*I and *EcoR*I and the *mgsD* fragment was ligated into pEKEx3 under control of the IPTG-inducible *tac* promoter, resulting in pEKEx3 *mgsD*, whose sequence identity was verified *via* restriction control and sequencing (Eurofins Genomics GmbH, Ebersberg, Germany) with primers mgsD1, mgsD2, mgsD3, and mgsD4.

The *manA* gene (cg0856), encoding mannose 6-phosphate isomerase (MPI), was amplified *via* PCR with primers manA_fwd and manA_rev from genomic DNA of *C. glutamicum* wild type (WT). The purified product was restricted with *Xba*I and *Kpn*I and cloned into the shuttle expression vector pVWEx1 under the control of the IPTG-inducible *tac* promoter. Sequence identity was verified *via* control digestions and sequencing using the primers mentioned above.

Gene *mgsD* was cloned into pVWEx1 *manA* by amplifying *mgsD* with primers mgsD-for (containing the ribosomal binding site of the *tuf* gene [cg0587] of *C. glutamicum*) and mgsD-rev from plasmid pEKEx3 *mgsD*. Plasmid pVWEx1 and the resulting PCR product were cut with *Kpn*I and purified *mgsD* was cloned into the cut plasmid *via* ligation. The resulting plasmid was sequenced with primers mgsD5, mgsD6, and mgsD7 to check sequence identity and correct orientation of *mgsD*.

### Determination of Specific Mannose 6-Phosphate Isomerase Activities

Determination of specific MPI activities was performed as described before (Sasaki et al., 2011). In brief, cells were grown in CGXII minimal medium with the respective carbon source(s) and harvested by centrifugation (4,200 × g, 10 min, 4°C). Cells were washed twice in 8 ml extraction buffer [2 mM dithiothreitol, 0.1 mM ethylenediaminetetraacetic acid, 20 mM KCl, 20 mM MgCl_2_, 5 mM MnSO_4_, and 100 mM tris(hydroxyethyl)aminomethane (TRIS), pH was adjusted to 7.5 with 2 M HCl] and resuspended in 1 ml extraction buffer. Before performing the assay, cells were disrupted in a Precellys 24 tissue homogenizer (Bertin Technologies SAS, Montigny-le-Bretonneux, France) for three rounds (45 s each, 6,500 × g, 4°C) with intermitted cooling on ice. The cell lysate was centrifuged (12,100 × g, 20 min, 4°C) and the supernatant was used for the assay in a final volume of 1 ml at 37°C. The assay contained 1 U glucose 6-phosphate dehydrogenase from yeast (Roche Diagnostics GmbH, Mannheim, Germany), 0.7 U phosphoglucose isomerase from yeast (Roche Diagnostics GmbH), 5 mM MgSO_4_, 25 mM TRIS-HCl (pH 7.5), 1 mM NADP^+^, 1 mM mannose 6-phosphate, and 100 µl of the respective (diluted) supernatant.

### Quantification of Substrates and Products

To quantify the substrates glucose and mannose and the product MG, 1 ml of cell suspension was harvested from the respective cultures by centrifugation (12,100 × g, 5 min, RT). Supernatants were either frozen and stored at −20°C or directly used for further analysis. Quantification of glucose, mannose, and mannosylglycerate was performed with an Agilent 1200 series apparatus (Agilent Technologies, Santa Clara, CA, United States) equipped with an organic acid/sugar column and precolumn (organic-acid resin; 300 × 8 mm; from CS Chromatographie, Langerwehe, Germany). Isocratic chromatography was realized with a mobile phase of 5 mM H_2_SO_4_ and a constant flow rate of 0.8 ml min^−1^ at 60°C. Detection occurred with a variable wavelength detector and a refractive index detector. Quantification of the analytes was conducted by using an eight-point calibration curve for each of the components (glucose, mannose, MG) as an external reference standard. MG was purchased as firoin from *Rhodothermus marinus* (Sigma Aldrich Chemie GmbH, Taufkirchen, Germany). The detection limit of the applied analytical system for MG in cell extracts was determined to be around 0.5 mM.

Intracellular MG concentrations were determined by harvesting 5 ml cell suspension from the cultures, centrifugation (4,200 × g, 20 min, RT), and resuspending the cell pellet in 1 ml 0.9% (w v^−1^) NaCl. Cells were disrupted in a Precellys 24 tissue homogenizer (Bertin Technologies SAS, Montigny-le-Bretonneux, France) for three rounds (45 s each, 6,500 × g, 4°C) with intermitted cooling at 4°C. The lysate was centrifuged (12,100 × g, 20 min, 4°C) and the supernatant was used for HPLC analysis, as described above.

Extracellular MG was determined by taking 1 ml samples from the culture medium, centrifugation at 12,100 × g, 5 min, RT, and directly using the supernatant for HPLC analysis as described above. The intracellular MG content was estimated using the following correlations:

(i) OD_600_ = 0.30 g_CDW_ L^−1^ (Blombach et al., 2007b).
(ii) 1 mg CDW = 2 µl cytoplasmic volume (Gutmann et al., 1992).

### Extraction of Mannosylglycerate and Bacterial-Milking-Like Experiment

MG was extracted from the cells by applying a cold water shock with ultrapure water (HiPerSolv CHROMANORM®, VWR International GmbH, Darmstadt, Germany) chilled to 4°C. This was done by harvesting 5 ml of the cell suspension at the respective time point, centrifugation (4,200 × g, 20 min, RT), resuspending the cell pellet in 5 ml ice-cold water, and incubating for 30 min on ice. After incubation, the cell suspension was centrifuged and samples were taken from the supernatant for extracellular MG analysis *via* HPLC as described above. To determine the amount of MG that remained intracellularly after the cold water shock, the cell pellet was treated as described above. It is noteworthy to mention that the viability of the cells was not affected by the cold water shock as the number of colony-forming units on 2xYT agar medium was the same before and after the cold shock treatment.

For a bacterial-milking-like experiment, cells were cultivated as described above with glucose and mannose as carbon sources. After 24 h, the complete content of a shaking flask was centrifuged and cells were resuspended in 20 ml of ice-cold ultrapure H_2_O and incubated for 60 min on ice. Afterward, the suspension was centrifuged again, and the supernatant was collected and stored at 4°C. The cell pellet was resuspended in 2 ml 0.9% (w v^−1^) NaCl solution and used to inoculate fresh medium. Cells were grown for 24 h at 30°C with 130 rpm shaking followed by another round of extraction with the supernatant from the first round of extraction. This procedure was repeated several times and the ice-cold supernatant (extract) was used repeated times to release MG from the cells, which led to the accumulation of MG.

## Results

### *C. glutamicum* WT Does Not Produce Mannosylglycerate, Does Not Metabolize It, and Is Not Inhibited by Mannosylglycerate

**FIGURE 2:**
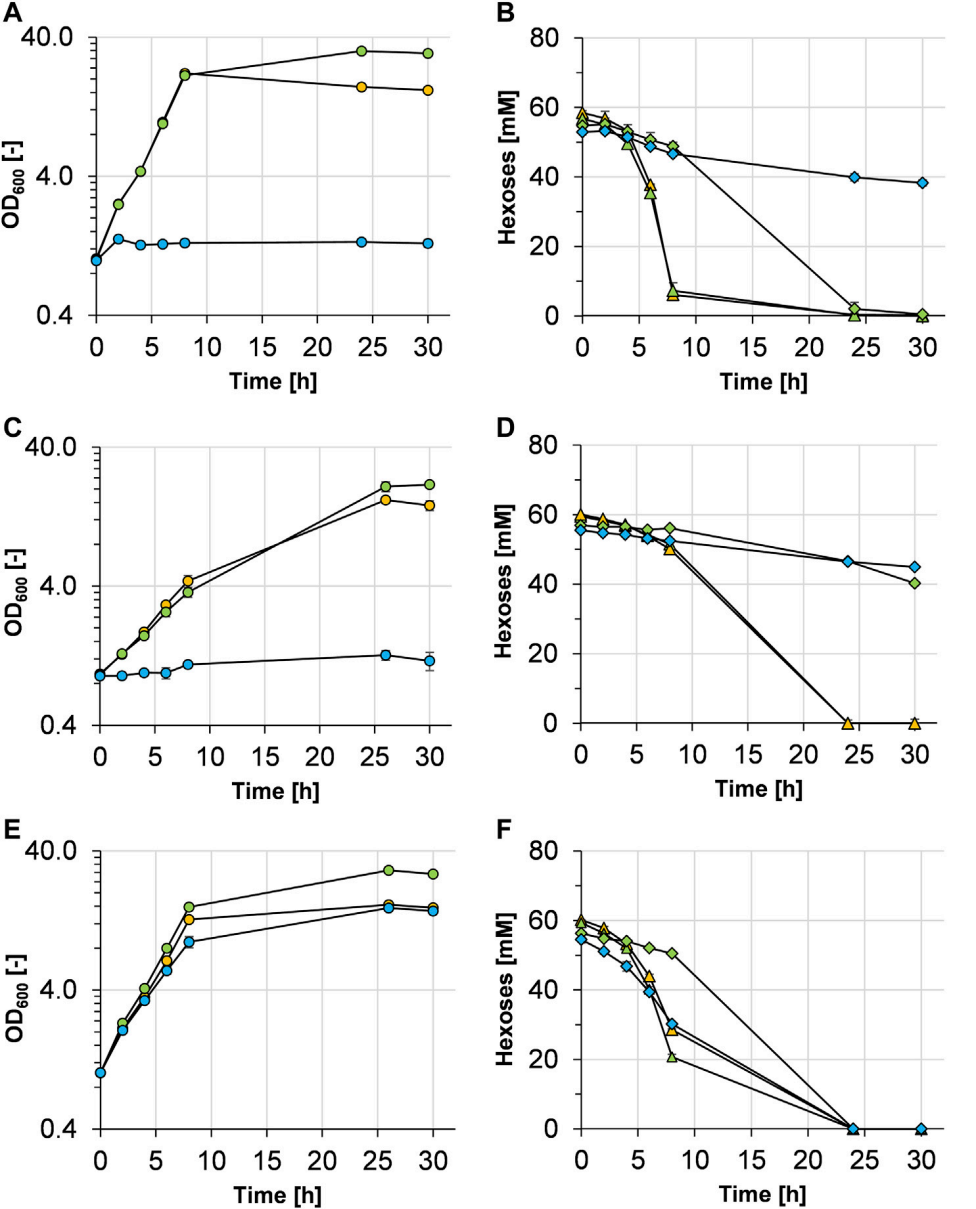
Growth and hexose (glucose and/or mannose) consumption of *C. glutamicum* WT **(A,B)**, *C. glutamicum* (pEKEx3 *mgsD*) **(C,D)**, and *C. glutamicum* (pVWEx1 *manA*) **(E,F)** grown in CGXII minimal medium containing either 1% glucose (orange symbols), 1% glucose plus 1% mannose (green symbols), or 1% mannose (blue symbols). The growth of the three strains is represented by colored circles in **(A,C,E)**. Glucose and mannose consumption in **(B,D,F)** is represented by triangles and diamonds and corresponds to the colors given in the growth diagrams, respectively. Experiments have been performed in triplicate and the standard deviation is given as error bars.

In the first experiments, *C. glutamicum* WT was cultivated in minimal medium with either glucose (10 g L^−1^), glucose plus mannose (10 g L^−1^ each), or mannose (10 g L^−1^) as carbon and energy source(s) (Figure 2A). With glucose and with a mixture of glucose and mannose, *C. glutamicum* WT grew with similar growth rates of 0.41 ± 0.01 and 0.40 ± 0.01 h^−1^, respectively. Due to the higher amount of carbon, the final OD_600_ of the cultures with glucose plus mannose was higher compared with that of the cultures with glucose alone. The WT strain was not able to grow at all with mannose as a sole carbon source (Figure 2A), although the mannose concentration in the medium slightly but steadily decreased over time (Figure 2B) with a rate of about 0.65 ± 0.02 mmol mannose g_CDW_^−1^ h^−1^). When both substrates were supplied, both carbon sources were metabolized; however, glucose was preferentially taken up and mannose was mainly metabolized after glucose was depleted (Figure 2B), indicating at least partially sequential metabolization of the two sugars.

Cells and supernatants of *C. glutamicum* WT cultures characterized above were then analyzed for the presence of MG. HPLC analysis of the intracellular and extracellular samples revealed that there was no MG detectable, suggesting that *C. glutamicum* natively is not able to synthesize MG.

To test whether MG exhibits negative effects on the growth of and whether MG is metabolized by *C. glutamicum*, the WT strain was inoculated and incubated in minimal medium with glucose (10 g L^−1^) and different concentrations of MG (6–24 mM) and the growth and MG concentration were followed. Growth of *C. glutamicum* WT in medium with glucose and MG was the same as that with glucose alone (data not shown) and the MG concentrations in the medium remained constant during cultivations, indicating that *C. glutamicum* is not able to take up MG and use it as a carbon source. In accordance, there was no growth observable in cultivations in medium with MG and without glucose (data not shown).

### Overexpression of a Heterologous *mgsD* Gene Leads to Mannosylglycerate Production

With the aim of enabling MG production in *C. glutamicum*, the heterologous *mgsD* gene from *D. mccartyi* was ligated into plasmid pEKEx3 and *C. glutamicum* WT was transformed with the resulting plasmid pEKEx3 *mgsD*. The resulting strain *C. glutamicum* (pEKEx3 *mgsD*) was cultivated in minimal medium with glucose, glucose plus mannose, or mannose as carbon source(s) (Figure 2C). The growth rates of *C. glutamicum* (pEKEx3 *mgsD*) in medium with glucose and with glucose plus mannose were 0.21 ± 0.02 and 0.18 ± 0.02 h^−1^, respectively. These growth rates are considerably lower than those of the plasmid-free host strain (see above, Figure 2A). As expected from the results obtained with *C. glutamicum* WT (Figure 2A), *C. glutamicum* (pEKEx3 *mgsD*) as well did not grow with mannose as a sole carbon source, although the mannose concentration in the medium again slowly decreased (Figure 2D) with a rate of 0.40 ± 0.09 mmol mannose g_CDW_^−1^ h^−1^.

Cells and supernatants of the *C. glutamicum* (pEKEx3 *mgsD*) cultures containing glucose as a carbon source were analyzed for the presence of MG. Using HPLC analysis, 111 ± 16 mM MG (60 ± 9 mg g_CDW_^−1^) were detected intracellularly after 24 h of cultivation when cells were grown with 20 g glucose L^−1^. No MG was detectable in supernatants of these cultivations, indicating that MG manifested as an intracellular metabolite in *C. glutamicum* and was not exported into the medium under these conditions, at least not in detectable amounts.

### Overexpression of *manA* Leads to Growth of *C. glutamicum* on Mannose

In *D. mccartyi* and some other organisms, MG is synthesized in a single-step reaction by the bifunctional enzyme MgsD from the glycolysis intermediate 3-PG and the activated sugar GDP-Man (Empadinhas et al., 2004; Löffler et al., 2013). Since GDP-Man can be formed from mannose in only one or two steps (see Figure 1), mannose represented an interesting substrate for a potential MG-producing *C. glutamicum* cell factory. Since *C. glutamicum* WT was not able to grow with mannose as a sole carbon source (Figure 2A), we aimed to exploit growth with metabolization of mannose by overexpressing the native *manA* gene. Overexpression of *manA* has been shown before to improve the growth of *C. glutamicum* strain R with mannose as a sole carbon source (Sasaki et al., 2011). Thus, plasmid pVWEx1 *manA* was constructed and used to transform *C. glutamicum,* resulting in strain *C. glutamicum* (pVWEx1 *manA*).

To evaluate the growth characteristics of *C. glutamicum* (pVWEx1 *manA*), it was cultivated in minimal medium containing glucose, glucose plus mannose, or mannose, and growth and substrate consumption were monitored (Figures 2E,F). With glucose and glucose plus mannose, *C. glutamicum* (pVWEx1 *manA*) showed slightly lower growth rates (0.32 ± 0.01 and 0.34 ± 0.01 h^−1^, respectively), when compared with that of *C. glutamicum* WT (0.40 ± 0.01 h^−1^). As observed with *C. glutamicum* WT, *C. glutamicum* (pVWEx1 *manA*) showed sequential metabolization of glucose and mannose (Figure 2F). However, in contrast to the WT strain, *C. glutamicum* (pVWEx1 *manA*) was able to grow with mannose as a sole carbon source (Figure 2E), showing a growth rate of 0.24 ± 0.01 h^−1^ and a mannose consumption rate of 2.49 ± 0.07 mmol mannose g_CDW_^−1^ h^−1^ within the first 7.5 h of incubation.

Overexpression of *manA* was evaluated by determination of the specific MPI activities in *C. glutamicum* WT and *C. glutamicum* (pVWEx1 *manA*) cells from the cultures characterized above (Figures 2A,E). As shown in Table 2, the specific MPI activities in extracts of *C. glutamicum* (pVWEX1 *manA*) were four- to sixfold higher in cells grown with glucose, with glucose plus mannose, and with mannose, when compared to the activities in extracts of *C. glutamicum* WT cells grown with glucose or with glucose plus mannose.

**TABLE 2:** Specific enzyme activities of mannose 6-phosphate isomerase (MPI) in cell extracts of *C. glutamicum* WT and *C. glutamicum* (pVWEx1 *manA*) cells, harvested during mid-exponential phase with either glucose, glucose plus mannose, or mannose as carbon source(s).

Strain	Specific MPI activity U (mg protein)^−1^		
	1% glucose	1% glucose plus 1% mannose	1% mannose
*C. glutamicum* WT	0.07 ± 0.02	0.10 ± 0.08	n.g.
*C. glutamicum* (pVWEx1 *manA*)	0.39 ± 0.10	0.40 ± 0.10	0.38 ± 0.01

n.g., no growth on this substrate. Experiments have been performed in triplicate and the standard deviation is given.

Taken together, our results show that the plasmid-borne, native *manA* gene in *C. glutamicum* (pVWEx1 *manA*) is overexpressed and that *manA* overexpression and thus, higher MPI activities enable *C. glutamicum* WT to grow on mannose as a sole carbon source.

### Combined Overexpression of *manA* and *mgsD* Enhances Mannosylglycerate Production and a Cold Shock Releases Mannosylglycerate From the Cytosol

**FIGURE 3:**
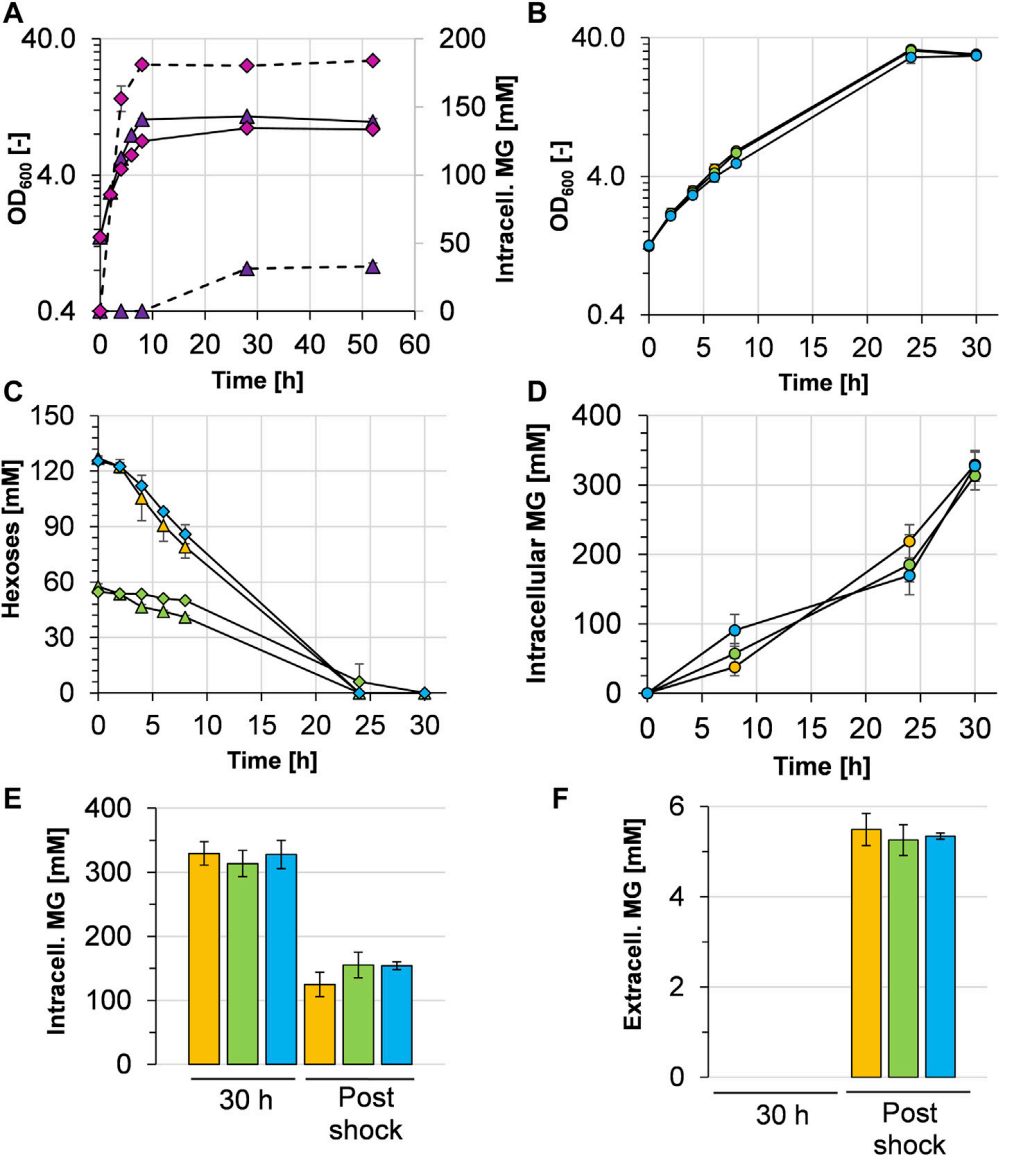
Growth characteristics and mannosylglycerate (MG) production behavior of *C. glutamicum* (pEKEx3 *mgsD*) (pVWEx1 *manA*) grown in different media. **(A)** Growth (solid lines) and intracellular MG concentration (dashed lines) in 2xYT complex medium with IPTG-induction (pink diamonds) and without IPTG-induction (purple triangles). **(B)** Growth and **(C)** hexose (glucose and mannose) consumption of *C. glutamicum* (pEKEx3 *mgsD*) (pVWEx1 *manA*) in CGXII minimal medium containing either 2% glucose (orange symbols), 1% glucose plus 1% mannose (green symbols), or 2% mannose (blue symbols) with the corresponding intracellular **(D)** and extracellular **(E)** MG concentrations, the latter upon cold shock with ultrapure H_2_O. Before the cold shock, extracellular MG concentrations were below the detection limit. Growth in **(B)** is represented by closed circles; glucose and mannose concentrations in **(C)** are represented by triangles and diamonds, respectively. Experiments were conducted in biological triplicates and the standard deviation is given as error bars.

With the aim of facilitating MG production from mannose as a carbon source, *C. glutamicum* (pEKEx3 *mgsD*) was transformed with plasmid pVWEx1 *manA*. The capacity of the resulting strain *C. glutamicum* (pEKEx3 *mgsD*)(pVWEx1 *manA*) to produce MG was investigated first by cultivations in a 2xYT complex medium (Figure 3A). When P_*tac*_-based expression of *mgsD* and *manA* was not induced with IPTG, *C. glutamicum* (pEKEx3 *mgsD*) (pVWEx1 *manA*) grew to a maximal OD_600_ of about 11 and accumulated a maximum of 33 ± 3 mM (18 ± 1 mg g_CDW_^−1^) of intracellular MG, which was probably due to leaky *mgsD* and *manA* expressions based on P_*tac*_. With the induction of IPTG, the strain grew to a slightly lower maximal OD_600_ of about 9 and accumulated up to 184 ± 2 mM (99 ± 1 mg g_CDW_^−1^) of intracellular MG. Importantly, the intracellular MG concentration remained stable in the stationary phase for ca. forty-two hours (Figure 3A), corroborating our previous conclusion that MG is not metabolized by *C. glutamicum* (see above).

To evaluate MG production in defined minimal medium, *C. glutamicum* (pEKEx3 *mgsD*) (pVWEx1 *manA*) was cultivated with 1% (w/v) glucose, 1% (wt/vol) glucose plus 1% (wt/vol) mannose, and 1% (wt/vol) mannose (Figure 3B). The growth rates between t = 0 and t = 8 h were 0.17 ± 0.01 h^−1^ with glucose, 0.17 ± 0.01 h^−1^ with glucose plus mannose, and 0.15 ± 0.01 h^−1^ with mannose. The respective consumption rates were 4.12 ± 0.43 mmol glucose g_CDW_^−1^ h^−1^ within the first 8 h of incubation, 1.49 ± 0.09 mmol glucose g_CDW_^−1^ h^−1^, and 0.41 ± 0.10 mmol mannose g_CDW_^−1^ h^−1^ and 4.49 ± 0.54 mmol mannose g_CDW_^−1^ h^−1^, respectively. The substrates (about 120 mM in every culture) were consumed within 24 h (Figure 3C), and the intracellular MG concentrations raised steadily (Figure 3D). After 30 h, the glucose-grown cells intracellularly accumulated 329 ± 18 mM, the glucose plus mannose-grown cells 314 ± 21 mM and the mannose-grown cells 328 ± 21 mM (Figure 3E), corresponding to 177 ± 10, 168 ± 11, and 176 ± 12 mg MG g_CDW_^−1^, respectively. However, analysis of MG in the culture supernatants revealed that the *C. glutamicum* (pEKEx3 *mgsD*) (pVWEx1 *manA*) cells under these conditions did not release MG in detectable amounts into the medium (data not shown). In summary, the results obtained with *C. glutamicum* (pEKEx3 *mgsD*) (pVWEx1 *manA*) reflect that mannose can substitute for glucose as a carbon source for intracellular MG production.

For biotechnological production processes, it is always of interest when the desired product can be harvested from culture broth. To enable the release of MG from the *C. glutamicum* cells into the medium, an osmotic temperature shock (cold water shock) with 4°C cold ultrapure water followed by 30 min incubation on ice was applied. As shown in Figure 3E, this treatment lowered the intracellular MG concentration by about 51–62%, leaving 125 ± 19, 155 ± 20, and 154 ± 6 mM in the cytosol of *C. glutamicum* (pVWEx1 *manA*) (pEKEx3 *mgsD*), respectively. Moreover, the cold water shock resulted in a release of MG from the cytosol into the medium, which led to extracellular MG concentrations of 5.5 ± 0.4 mM (1.48 g L^−1^) for glucose-grown cells, 5.3 ± 0.3 mM (1.42 g L^−1^) for glucose plus mannose-grown cells, and 5.3 ± 0.1 mM (1.42 g L^−1^) for mannose-grown cells (Figure 3F). The yields thus were in the range of about 1.45 g MG per 20 g of sugar, corresponding to 0.073 g MG g_sugar_^−1^ or 7.3%.

### Constructing a One-Plasmid System for Mannose Utilization and Mannosylglycerate Production and Applying It for Cumulative Mannosylglycerate Production

**FIGURE 4:**
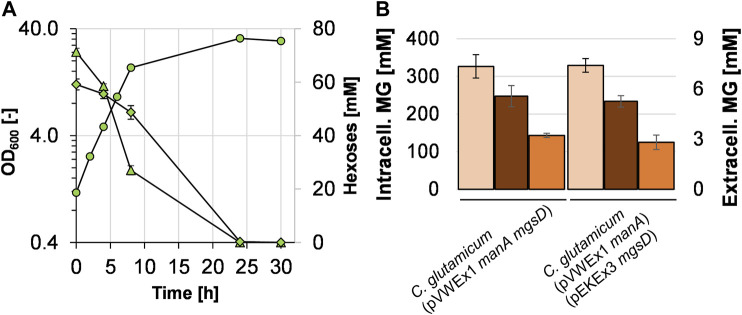
Growth of *C. glutamicum* (pVWEx1 *manA mgsD*) with 1% glucose (triangles) and 1% mannose (diamonds) in CGXII minimal medium **(A)** and comparison of intracellularly accumulated MG (light red bars), released MG (dark bars), and remaining intracellular MG (orange bars) obtained with *C. glutamicum* (pVWEx1 *manA*) (pEKEx3 *mgsD*) under the same conditions **(B)**.

To construct a one-plasmid system for combining efficient mannose utilization and MG production, the *mgsD* gene was cloned into the pVWEx1 *manA* plasmid and the resulting plasmid pVWEx1 *manA mgsD* was used to transform *C. glutamicum.* The resulting strain *C. glutamicum* (pVWEx1 *manA mgsD*) was cultivated in minimal medium with glucose plus mannose (Figure 4A). The growth rate of *C. glutamicum* (pVWEx1 *manA mgsD*) was significantly higher when compared to that of the strain carrying two plasmids, *C. glutamicum* (pVWEx1 *manA*) (pEKEx3 *mgsD*) (0.32 ± 0.01 vs. 0.17 ± 0.01 h^−1^, see also Figure 3B). Both strains showed very similar MG production capacities (Figure 4B); the intracellular MG concentration in *C. glutamicum* (pVWEx1 *manA mgsD*) was determined to be 327 ± 31 mM (= 175 ± 17 mg MG g_CDW_^−1^), compared to 314 ± 21 mM in the two-plasmid-strain. After cold water shock, the released MG reached 5.6 ± 0.6 mM, leaving 143 ± 6 mM of intracellular MG in the cytosol, compared to 5.3 ± 0.34 mM extracellularly and 155 ± 20 mM intracellularly with *C. glutamicum* (pVWEx1 *manA*) (pEKEx3 *mgsD*). These values correspond to recoveries after cold water shock of about 56% for the one-plasmid system and 51% for the two-plasmid system.

**FIGURE 5:**
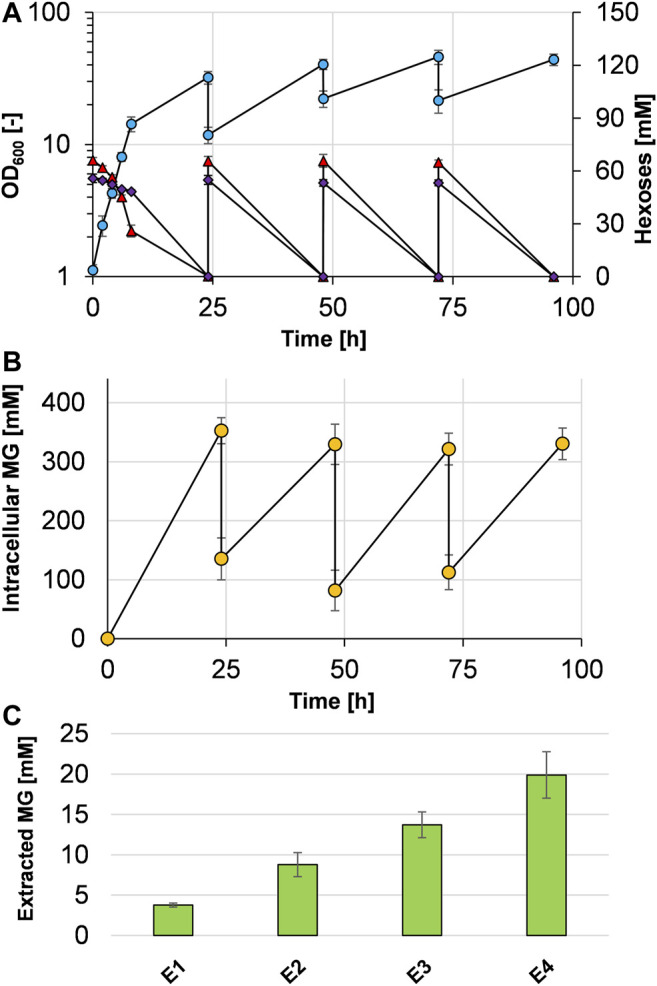
Bacterial milking experiment with *C. glutamicum* (pVWEx1 *manA mgsD*) in CGXII minimal medium with 1% glucose and 1% mannose. Cells were cultivated 24 h in repeated cycles and growth (blue circles), mannose (purple diamonds), and glucose (red triangles) concentrations are given **(A)**. After 24 h of cultivation, the cells were harvested by centrifugation and shocked with ultrapure ice-cold water, leading to the release of MG from the cytosol, determined by analysis of intracellular MG concentrations **(B)**. The extract solution was collected by centrifugation and stored at 4°C; the cell pellet was used to inoculate the fresh medium. The procedure was repeated three times; the stored extract was used for another round of extraction 24 h later. This procedure led to stepwise accumulation of MG in the extract **(C)**. Concentrations of the released and cumulated MG that were measured in the supernatant after the single extraction steps (E1–E4) are shown as bars. Experiments were conducted in biological triplicates and the standard deviation is given as error bars.

To test whether cells can be recycled and applied for repeated cycles of cultivation and production, we set up an experiment similar to what has previously been termed *bacterial milking* (Sauer and Galinski, 1998). For that purpose, *C. glutamicum* (pVWEx1 *manA mgsD*) was cultivated for 24 h in minimal medium with mannose and glucose (Figure 5A) and cells were harvested and exposed to a cold water shock, which released MG from the cytosol. Afterward, these cells were used to inoculate fresh medium, the culture was incubated again for 24 h, and the cells were again subjected to cold water shock treatment. The initial OD_600_ dropped from ca. 32–12, which is due to the previous sampling for determination of OD_600_, of the substrates, and of intra- and extracellular MG. The growth-and-extraction cycle was repeated three times, leading to repeated increase and decrease in the intracellular MG concentration (Figure 5B) and stepwise increase in the MG concentration of the “milking extract” (Figure 5C). Four extractions (E1–E4 in Figure 5C) led to the release of 3.8 ± 0.3, 5.0 ± 1.2, 4.9 ± 0.1, and 6.2 ± 1.3 mM MG, respectively, which eventually summed up to 19.9 ± 2.9 mM MG (5.34 g L^−1^) in the extract solution. The final yield of this experiment was 5.34 g MG per 80 g of sugar, corresponding to 0.067 g MG g_sugar_^−1^ or 6.7%.

## Discussion

MG is a compatible solute that occurs mainly in (hyper)thermophilic organisms (Santos and da Costa, 2002; Borges et al., 2014) and red algae of the order Ceramiales (Kremer, 1980) and represents an interesting product for microbial production due to its superior protective properties on protein structures *in vivo* and *in vitro*. Therefore, it might have great potential to promote the development of new drugs for protein-misfolding diseases, such as Parkinson’s and Alzheimer’s disease (Faria et al., 2013; Jorge et al., 2016). However, in contrast to other compatible solutes like ectoine (Fallet et al., 2010; He et al., 2015; Zhao et al., 2019) or trehalose (Schiraldi et al., 2002; Song et al., 2016), whose production has been exploited intensely, the knowledge on the production of MG is more restricted.

*C. glutamicum* not only is a suitable host for MG production due to the vast set of established engineering tools and its genetic accessibility (Becker and Wittmann, 2015; Pérez-García and Wendisch, 2018) but also does not take up and metabolize MG (as shown here) like other industrially relevant organisms, such as *E. coli* and *Bacillus subtilis* (Sampaio et al., 2004). *C. glutamicum* has been engineered to produce the native compatibles solutes trehalose (Carpinelli et al., 2006) and L-proline (Jensen and Wendisch, 2013) and the nonnative compatible solutes α-D-glucosylglycerol (Roenneke et al., 2018), L-pipecolic acid (Pérez-García et al., 2019), ectoine/hydroxyectoine (Gießelmann et al., 2019), and very recently also MG (Tian et al., 2020). In the latter work, MG production has been reported from glucose and mannose plus glycerate and authors applied MG synthase from *Rhodothermus marinus* DSM 4252 and additionally overexpressed the genes *nahK* from *Bifidobacterium longum*, encoding N-acetylhexosamine 1-kinase, *manB*, encoding phosphomannomutase from *E. coli*, and *manC*, encoding mannose 1-phosphate guanylyl transferase from *E. coli* to convert glucose/mannose to MG (Tian et al., 2020). This strain produced 2.1 g MG L^−1^ from 20 g glucose and 5 g glycerate L^−1^, 5.5 g MG L^−1^ from 20 g mannose and 5 g glycerate L^−1^, and 7.2 g MG L^−1^ from mixed sugar substrates containing 30.6 g mannose L^−1^, 5.1 g fructose L^−1^, 5.0 g glucose L^−1^, and 10 g glycerate L^−1^ in a high cell density fermentation processes with an inoculation OD_600_ of 25. Interestingly, production from mannose and glycerate was 2.6-fold higher than from glucose and glycerate. In the data presented in this work, production from glucose, from glucose plus mannose, and from mannose alone yielded similar results, not highlighting one over the other carbon source. This might have to do with the fact that 0.5% glycerate was supplemented by Tian et al. and served as an additional carbon source.

Besides *C. glutamicum*, also *S. cerevisiae* has been genetically modified to produce MG (Faria et al., 2018). The MG-producing *S. cerevisiae* strain (over)expressed the heterologous *mgsD* gene of *D. mccartyi* and the homologous PMI40 (encoding mannose-6-phosphate isomerase) and PSA1 (encoding GDP-mannose pyrophosphorylase) genes, resulting in an accumulation of up to 16 mg MG g_CDW_^−1^. Compared to this intracellular MG content, the herein presented 177 mg g_CDW_^−1^ in *C. glutamicum* is more than tenfold higher. The respective value of the *C. glutamicum* strain constructed by Tian et al. (2020) accumulated even more than 400 mg MG g_CDW_^−1^; however, this was produced from a mixture of several sugars and the direct precursor glycerate at quite high concentrations. Up to about 86 mg MG g_CDW_^−1^ and a final titer of 1.7 g MG L^−1^ have been produced in a fed-batch fermentation in a complex medium using a trehalose-deficient mutant of *Thermus thermophilus* RQ-1 (Egorova et al., 2007), a thermophilic bacterium that natively forms MG in response to osmotic stress (Nunes et al., 1995). Commercially, MG has been reported to be produced by bitop AG (Witten, Germany) *via* fermentation of a natural producer, *Rhodothermus marinus*, but this resulted in low titers and high production costs (Faria et al., 2018).

Since MG by the deployed MG synthase from *D. mccartyi* is synthesized from 3-phosphoglycerate and the activated sugar GDP-mannose, it was logical to test mannose as a sole and additional carbon source for MG production. However, the basis strain *C. glutamicum* ATCC 13032 naturally does not grow on mannose (Figure 2A). In contrast, *C. glutamicum* R at least showed minimal growth with mannose as a sole carbon source (Sasaki et al., 2011). As in *C. glutamicum* R (Sasaki et al., 2011), overexpression of the native *manA* gene led to efficient mannose metabolization in *C. glutamicum* ATCC 13032, thereby enabling exploitation of this carbon source for MG production. Determination of specific mannose 6-phosphate isomerase activities in *C. glutamicum* (pVWEx1 *manA*) demonstrated successful overexpression of *manA* and resulted in about 8-fold increased specific activities on all tested carbon sources/mixtures during the exponential growth phase, when compared to *C. glutamicum* WT. It has been shown that the EII subunit of the phosphoenolpyruvate- (PEP-) dependent phosphotransferase system (PTS) for glucose (encoded by *ptsG*) has very limited transport capacities for mannose besides its main substrate glucose (Moon et al., 2007). Further research indicated that both the fructose and glucose PTS are involved in mannose uptake (Sasaki et al., 2011). These authors also observed increased mannose uptake of a *manA*-overexpressing strain when a mixture of mannose and glucose was present compared with mannose as a sole carbon source. Glucose in all cases was strongly preferred over mannose (Sasaki et al., 2011), which could be observed in this work as well. Nevertheless, the exact mechanism of mannose uptake in *C. glutamicum* remains unclear and requires further research. So far, mannose is a neglected carbon source for cultivations of *C. glutamicum* and its use is limited to those products whose precursor is GDP-mannose and to which mannose lies in close spaciotemporal vicinity, such as GDP-L-fucose (Lee et al., 2009; Chin et al., 2013), mannosyl-oligosaccharides, and MG (Tian et al., 2020). Interestingly, the overexpression of *manA* in a GDP-L-fucose-producing *C. glutamicum* strain showed negative effects on the formation of the product (Chin et al., 2013). In contrast to Tian and coworkers, we aimed to produce MG from glucose and mannose as sole carbon source(s), without supplementations like glycerate. For that purpose, *manA*, enabling mannose utilization, and *mgsD*, enabling MG synthesis, were combined in a two-plasmid system, showing solid production of MG, which accumulated intracellularly. When *manA* and *mgsD* genes were combined in one plasmid, MG production behavior remained similar; however, the resulting strain showed faster growth, which most likely is connected to the omitted spectinomycin for maintenance of the second plasmid.

When comparing the MG-producing strains *C. glutamicum* (pEKEx3 mgsD) and *C. glutamicum* (pEKEx3-mgsD) (pVWEx1 manA), the growth rates on glucose medium were almost the same (Figures 2C, 3B; 0.21 ± 0.02 vs. 0.17 ± 0.01 h^−1^). The former strain accumulated intracellularly 111 mM MG with glucose as a substrate, and the latter accumulated 329 mM MG, indicating that additional overexpression of manA in fact is very favorable for MG synthesis. This is probably due to the fact that the ManA protein (MPI) is very favorable in converting fructose-6-phosphate to mannose-6-phosphate (see Figure 1) and thus provides this precursor of MG synthesis very efficiently.

Extraction of MG from the cytosol into the medium was accomplished with a cold water shock. This method principally was shown before to extract isotopically labeled MG from the *E. coli* cytosol (Sampaio et al., 2003). Preliminary experiments indicated that mainly the temperature is responsible for the release, since a physiological ice-cold sodium chloride solution extracted MG to a small percentage, whereas ultrapure water at room temperature did not lead to a release of detectable amounts of MG (data not shown). In contrast to the work of Sampaio and colleagues, where almost 100% of radioactively labeled MG could be released from the *E. coli* cytosol into the medium (Sampaio et al., 2003), we here report on the release of up to 62% of MG release from the *C. glutamicum* cytosol. This difference might have to do with the transport capacities for MG, which most likely differs in both organisms. Interestingly, the release of MG from the *C. glutamicum* cytosol into the medium has been reported before to occur without application of a shock leading to the release of about 48% of the MG into the medium (Tian et al., 2020). Since the intracellular concentrations in these strains were higher than in the herein reported ones, it might be speculated that intracellular concentrations of MG in *C. glutamicum* have a certain threshold and upon reaching it, MG leaks out of the cell. In contrast to the export of MG, which has not been studied in detail, the import of MG has been elucidated for *E. coli*, which in contrast to *C. glutamicum* is able to grow with MG as a sole carbon source. In *E. coli*, uptake is mediated by the PEP-dependent PTS with protein MngA as an MG-inducible specific EIIABC complex (Sampaio et al., 2004).

Since the production of compatible solutes with native producers has been achieved successfully before by the so-called *bacterial milking* (Sauer and Galinski, 1998; Egorova et al., 2007), *C. glutamicum* (pVWEx1 *manA mgsD*) was employed for a similar process. Alternating rounds of growth/production and osmotic shocks were used to extract the produced MG from the cells, eventually leading to 5.34 g MG L^−1^ after repeating the extraction three times. In principle, these cycles could be repeated several times more on a larger scale to increase the amount of extracted MG. Similar techniques have been applied before for the production of compatible solutes in their native hosts, e.g., ectoine/hydroxyectoine with *Halomonas elongata* (Sauer and Galinski, 1998; up to 4.1 g L^−1^) and MG with *Thermus thermophilus* RQ-1 (Egorova et al., 2007; 4.6 g L^−1^). Recombinant *C. glutamicum* cells grown under elevated osmotic pressure released L-pipecolic acid (about 2.8 g L^−1^) after an osmotic down-shock (Perez-Garcia et al., 2019), in principle also enabling bacterial milking with repeated cycles. However, the former two processes in contrast to the one that is described here required increased concentrations of sodium chloride of up to 30 g L^−1^, which is well known to act corrosively on steel reactors and thus might hamper industrial feasibility (Speidel, 1981; Chisti, 1992).

Sustainable production of biochemicals and pharmaceuticals is an intensively discussed and increasingly important topic (Meyer, 2011; Koenig et al., 2019; Matthews et al., 2019). Utilizing green substrates with as little impact on the environment as possible and leading production away from fossil-based resources nowadays has top priorities. Optimally, such sustainable bioprocesses use renewable and not food-carbohydrates-based substrates, to circumvent the *food vs. fuel* debate (Inderwildi and King, 2009). One source for such microbial substrates is hexose and pentose sugars derived from lignocellulose-based materials, which are not intended for food or feed production and often arise from processing plant materials in biorefineries (Valdivia et al., 2016). Conversion of the input lignocellulosic biomass, which is heterogenous in plant origin and composition, results in side streams as byproducts, such as spent sulfite liquor (SSL), a waste stream in pulp and paper manufacturing processes. SSL contains mannose (up to more than 40% of the total sugars), glucose (up to more than 20%), and xylose (up to 20%) as the main carbohydrates, depending on the wood type used in fluctuating proportions (Nigam, 2001; He and Chen, 2020; Hoheneder et al., 2021). However, although SSL in general contains potential inhibitors for bacterial growth (Al-Rudainy et al., 2017), *C. glutamicum* carrying our plasmid pVWEx1-manA has recently been shown to grow in minimal medium containing 20% of ultrafiltrated SSL from softwood pulping, consuming completely both glucose and mannose (Sinner et al., 2021). Moreover, Perez-Gracia et al. (2021) used synthetic SSL, containing glucose, mannose, and xylose, as a substrate for a riboflavin-producing *C. glutamicum* strain with the plasmid-bound expression of *manA* and *xylAB* genes, the latter enabling xylose utilization. Thus, it might be attractive to use *C. glutamicum* (pVWEx1 *manA mgsD*) constructed here for MG production from SSL, a green substrate, that is available in large quantities from lignocellulosic biomass.

## Conclusion

Taken together, we showed that *C. glutamicum* can be engineered to produce the compatible solute MG by expressing *mgsD* from *D. mccartyi* from glucose and from glucose plus mannose and when the native *manA* is overexpressed from mannose alone as well. The intracellular MG accumulated up to 177 mg MG g_CDW_^−1^ and up to 62% thereof could be released with a cold water shock, resulting in up to 1.48 g MG L^−1^. A *bacterial milking*-like experiment with repeated cycles of production and extraction led to 5.34 g MG L^−1^. Our results provide valuable information to develop *C. glutamicum* as a promising host for industrial production of MG from waste streams containing glucose and/or mannose, such as SSL derived from processed lignocellulosic biomass.

## Data Availability

The original contributions presented in the study are included in the article/Supplementary Material; further inquiries can be directed to the corresponding author.
